# Treatment of Hypothyroid Patients With L-Thyroxine (L-T4) Plus Triiodothyronine Sulfate (T3S). A Phase II, Open-Label, Single Center, Parallel Groups Study on Therapeutic Efficacy and Tolerability

**DOI:** 10.3389/fendo.2019.00826

**Published:** 2019-11-29

**Authors:** Ferruccio Santini, Giovanni Ceccarini, Caterina Pelosini, Monica Giannetti, Ilaria Ricco, Giorgia Querci, Enzo Grossi, Giorgio Saponati, Paolo Vitti

**Affiliations:** ^1^Endocrinology Unit, University Hospital of Pisa, Pisa, Italy; ^2^Fondazione Bracco, Milan, Italy; ^3^ISPharm CRO, Lucca, Italy

**Keywords:** hypothyroidism, L-Thyroxine, substitutive therapy, thyroid hormone metabolism, 3,5,3′-triiodothyronine sulfate

## Abstract

Sodium salt of levothyroxine (L-T4) is the treatment of choice of hypothyroidism. Yet, L-T4 monotherapy produces supoptimal 3,5,3′-triiodothyronine (T3)/T4 ratio in serum, as compared to normal subjects, and a minority of hypothyroid individuals on L-T4 complain for an incomplete well-being. Orally administered 3,5,3′-triiodothyronine sulfate (T3S) can be converted to T3 in humans, resulting in steady-state serum T3 concentrations for up to 48 h. In this study (EudraCT number 2010-018663-42), 36 thyroidectomized hypothyroid patients receiving 100 (group A), 125 (group B), or 150 μg (group C) L-T4 were enrolled in a 75 days study in which 25 μg L-T4 were replaced by 40 μg of T3S. A significant, progressive reduction in mean FT4 values was observed, being the largest in the group A and the smallest in group C, while no relevant variations in FT3 and total T3 serum values were observed in the three groups. TSH serum levels increased in all groups, the highest value being observed in group A. Lipid parameters did not show clinically significant changes in all groups. No T3S-related changes in the safety laboratory tests were recorded. No adverse event was judged as related to experimental treatment, and no patient discontinued the treatment. Twelve patients judged the L-T4+T3S treatment better than L-T4 alone, while no patient reported a preference for L-T4 over the combined treatment.

In conclusion, the results of this study indicate that a combination of L-T4+T3S in hypothyroid subjects may allow mainteinance of normal levels of serum T3, with restoration of a physiological FT4/FT3 ratio and no appearance of adverse events. Further studies are required to verify whether the LT4+T3S chronic combined treatment of hypothyroidism is able to produce additional benefits over L-T4 monotherapy.

## Introduction

Hypothyroidism is one of the most common endocrine disorders (1), developing as a consequence of impaired action of thyroid hormones on target tissues (2). In most instances hypothyroidism is due to insufficient secretion of thyroid hormone, turning into reduced serum concentrations of thyroxine (T4) and triiodothyronine (T3). T4 is the main product of thyroid secretion, but needs to be converted to T3 in order to bind to thyroid hormone receptors. Only a lesser fraction of circulating T3 is produced directly by the thyroid whereas the majority derives from outer-ring deiodination of T4 in peripheral tissues (3). Beside deiodination, both T4 and T3 can be metabolized by alternate pathways, mainly sulfation and glucuronidation (4, 5).

Appropriate hormonal substitutive treatment is essential to reduce morbility and mortality of hypothyroid subjects (6). Even though T3 is the active molecule, administration of sodium salt of levothyroxine (L-T4) is the substitutive treatment of choice in hypothyroidism (7), since daily oral ingestion of L-T4 warrants stable serum T3 concentrations and prevents the non-physiologic surges of T3, which occur after L-triiodothyronine (L-T3) oral intake. Nevertheless, L-T4 monotherapy produces a low T3/T4 ratio in serum, as compared to normal subjects, since the amount of T3 directly secreted from the thyroid is lacking (8–11). Furthermore, a minority of hypothyroid individuals on L-T4 complain for an incomplete well-being and about symptoms that may be reconducted to a hypothyroid status, despite having their serum TSH within the normal range. Several studies have been conducted to explore the possibility of restoring physiological concentrations of thyroid hormones by administration of a combination of L-T4 and L-T3 in various proportion, but no clear advantages could be demonstrated over the standard L-T4 monotherapy (12, 13).

We have recently demonstrated that 3,5,3′-triiodothyronine sulfate (T3S) can be converted to T3 following oral administration in humans. T3S to T3 conversion was dose dependent and, after a single dose, resulted in steady-state serum T3 concentrations for up to 48 h, suggesting that T3S might represent a new agent to be combined with L-T4 for the treatment of hypothyroidism (14). The aim of this proof of principle study was to investigate the efficacy and safety of combined administration of L-T4+T3S in hypothyroid subjects for a prolonged period.

## Materials and Methods

The study was performed at a single center (Endocrinology Unit, University Hospital of Pisa, Italy). Project management, monitoring and reporting of the study were carried out by the Contract Research Organization (CRO) ISPharm srl, Via Dorati 117, Lucca, Italy. Data management and statistical analysis were carried out by the Studio Associato Airoldi, Cicogna e Ghirri, Via Manzoni 43, Milan, Italy. Laboratory tests were performed at the Pisa Hospital, except T3 sulfate assay, which was performed at CRB/Biology, Bracco Research Center, Via Ribes 5, Colleretto Giacosa (TO), Italy.

Description of T3S synthesis, composition of pharmaceutical form and hormonal assays have been previously reported (14).

The study was performed according to a phase II, open-label, uncontrolled, single center, parallel groups study design. Inclusion criteria were: written informed consent; outpatient of either sex, aged between 18 and 70 years; total thyroidectomy for any reason, on stable (at least 3 months) substitutive or TSH suppressive L-T4 therapy (daily dose: 100/125/150 μg); no evidence of endogenous hormonal production (thyroglobulin <5 ng/mL and undetectable thyroglobulin antibody); FT_4_, FT_3_, Total T3 (TT3) values within the normal ranges for euthyroid subjects reliability in terms of medication compliance and capability of understanding the study protocol procedures and timelines.

Exclusion criteria were: history or current evidence of cardiovascular diseases, e.g., congestive heart failure NYHA class >1, coronary artery disease, myocardial infarction, severe hypertension, cardiac arrhythmias; history, or current evidence of significant liver (i.e., AST/ALT higher than twice the upper limit of normal range) or renal (i.e., creatinine >2 mg/dl) failure, metabolic, or endocrine diseases (e.g., uncontrolled diabetes mellitus), or any other underlying medical condition that might interfere with the study; treatment with drugs affecting thyroid hormone absorption or metabolism; any medical condition or other circumstances which would significantly decrease the chances of obtaining reliable data, achieving study objectives, or completing the study and/or post dose follow-up examinations; immunocompromised patients; malignant diseases or any other disease with life expectancy <2 years; history of alcohol abuse, drug abuse, psychological, or other psychiatric diseases that could invalidate informed consent or limit the subject compliance with protocol requirements; artificial or parenteral feeding; allergy, sensitivity, or intolerance to study drugs and/or any of study drug formulation ingredients; pregnant or breastfeeding females, or females not practicing adequate contraceptive measures; patients unlikely to comply with the protocol or unable to understand the nature, scope and possible consequences of the study; patients who received any investigational drug within the last 3 months; employees of the study center (i.e., principal investigator, sub-investigator, study coordinators, other study staff, employees, or contractors of each), with direct involvement in the proposed study, as well as family members of the employees or the investigator.

Thirty-six Caucasian thyroidectomized outpatients were enrolled and assigned to one of 3 treatment groups (12 patients for each group) according to the ongoing L-T4 dose: 100, 125, or 150 μg daily, and named group A, B, and C, respectively. Patients were consecutively enrolled until each group achieved the required number. There were no blinding procedures. The baseline characteristics of enrolled patients are reported in Table 1.

**Table 1:** Patients' characteristics at study entry.

		**Group A (12 patients)**	**Group B (12 patients)**	**Group C (12 patients)**	**Overall (36 patients)**
Age (years)	Min–max	32.5–68.4	32.4–65.1	26.1–57.7	26.1–68.4
	Median	50.1	56.3	47.7	50.1
	Mean ± SD	50.8± 11.4	53.1 ± 9.2	46.1 ± 8.7	50.0 ± 10.0
Gender, *N* (%)	Female	11 (92)	5 (42)	9 (75)	25 (69)
	Male	1 (8)	7 (58)	3 (25)	11 (31)
Weight (Kg)	Min–max	554–95	68–110	58–102	54–110
	Median	66	78	84	75
	Mean ± SD	68 ± 13	81 ± 13	84 ± 13	78 ± 14
Height (cm)	Min–max	154–175	160–180	160–180	154–180
	Median	165	169	171	168
	Mean ± SD	165 ± 6	170 ± 6	170 ± 6	168 ± 7
BMI (Kg/m^2^)	Min–max	19.8–33.8	23.5–38.3	21.3–38.9	19.8–38.9
	Median	23.9	26.9	29.1	26.8
	Mean ± SD	25.2 ± 4.4	28.1 ± 4.3	29.0 ± 4.7	27.4 ± 4.7
Reason for thyroidectomy, *N* (%)	Thyroid cancer	9 (75)	10 (92)	9 (75)	29 (81)
	Nodular goiter	3 (25)	1 (8)	3 (25)	7 (19)
Time since thyroidectomy (years)	Min–max	0.6–15.4	1.5–31	0.4–11.3	0.4–31
	Median	3.0	5.9	4.4	5.0

On average, T4 and T3 are secreted by the thyroid in a molar ratio of about 15:1, corresponding to 100 μg T4 and 6 μg T3. The amount of T3 that is directly produced by the thyroid is about 20% of daily T3 production (30 μg), meaning that 24 μg T3 are produced by peripheral deiodination of T4. Based on these assumption, 25 μg of the L-T4 dose, representing the amount of T4 that by peripheral deiodination should provide 6 μg T3, was substituted with T3S. The dose of 40 μg T3S was selected on the basis of the previous study (13), as the lower dosage able to attain safe and putatively effective serum levels of FT3.

The L-T4 dose was therefore partially replaced by T3S, as follows:

**Table:** 

Group A	From 100 μg L-T4
	To 75 μg L-T4 +40 μg T3S
Group B	From 125 μg L-T4
	To 100 μg L-T4 + 40 μg T3S
Group C	From 150 μg L-T4
	To 125 μg L-T4 + 40 μg T3S

The investigational product was administered together with L-T4, in the morning, after at least 12 h fasting; food intake was restrained for 20 min post-dose. During the study the L-T4 dose remained unchanged, whereas the T3S dose was suitable for decrease or increase by steps of 20 μg daily (up to 100 μg maximum daily dose) based on to the hormonal status (FT_3_, FT_4_, TSH), the clinical findings and the investigator opinion.

**Figure 1:**
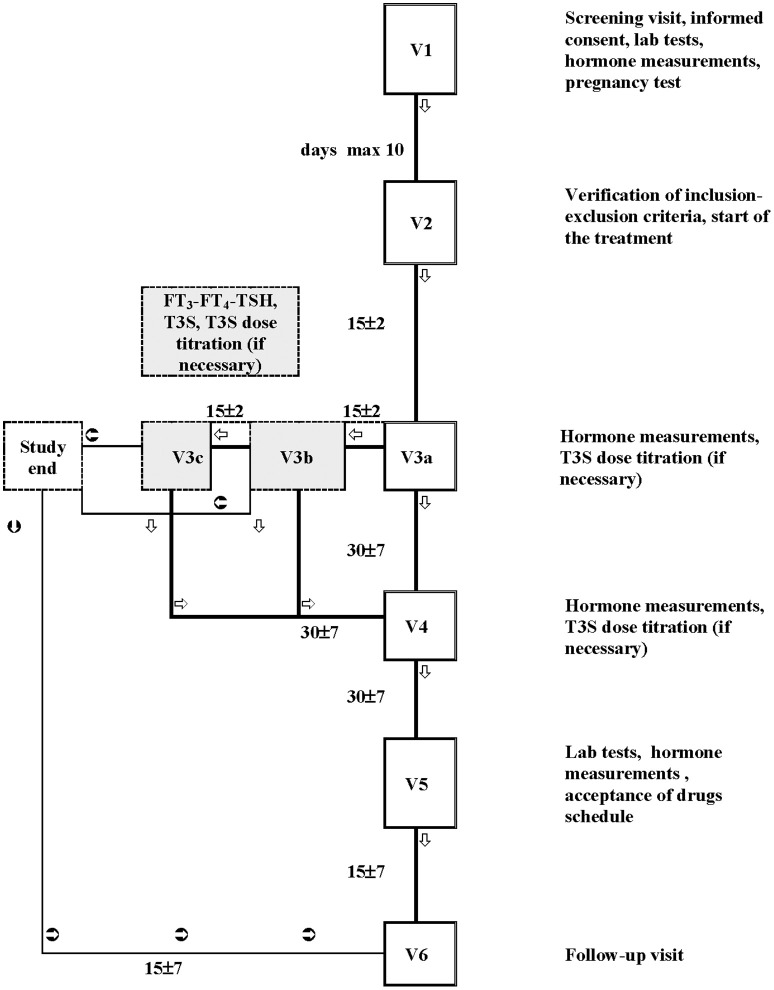
Flow chart of the study. The study plan included a screening visit (Visit 1), in which patients potentially eligible were checked for inclusion and exclusion criteria; Visit 2 was performed within 10 days from Visit 1 to confirm the compliance with the inclusion and the exclusion criteria; if confirmed, the L-T4 therapy schedule was changed to L-T4+T3S. The next visits (max 3 visits: V3a, V3b, and V3c) were performed every 15 days and were dedicated to T3S titration. If during the titration period the patients maintained (or attained) the metabolic control (i.e., hormonal parameters in the accepted range), the T4+T3S dosage remained unchanged until the next follow-up visit (Visit 4, 1 month after), when a T3S dosage change was allowed. A fifth visit (visit 5) was performed after a further month of therapy. If at the end of the titration period (visit 3c) patients did not attained the metabolic control, they were removed from the study. Intermediate visit(s) were arranged in case of adverse events or whenever judged suitable for patient safety by the investigator. A safety follow-up visit (visit 6) was arranged 15 (+ maximum 7) days after visit 5.

The study flow-chart is shown in Figure 1. After starting T3S, patients were visited every 15 days (for a maximum of 45 days) until the euthyroid state was achieved (titration period). The following control visits were performed monthly for 2 months. Routine hematology included measurement of: red blood cell count, white total and differential blood cell count, hemoglobin, hematocrit, and platelets count. Routine blood chemistry included measurement of: liver enzymes, creatinine, blood urea nitrogen, fasting plasma glucose, albumin, total protein, and electrolytes (sodium, chloride, and potassium).

The primary objective of the study was to investigate the feasibility of maintaining the euthyroid state -based on TT3, FT3, FT4, and TSH circulating levels in hypothyroid patients, by partially substituting the actual dose of L-T4 with T3S. The secondary objectives were: to assess the safety of T3S prolonged administration; to identify the optimal L-T4/T3S substitutive ratio; to compare the judgment of the patients on L-T4 vs. L-T4+T3S therapy; to evaluate the effects of the combined therapy on serum lipids profile.

The study protocol, patient information leaflet and informed consent document were approved by the reference Independent Ethics Committee (IEC) of the investigational study site (CEAVNO Comitato Etico Area Vasta Nord Ovest), all patients gave written informed consent in accordance with the Declaration of Helsinki. The study was registered at EudraCT with the study number 2010-018663-42.

## Statistical Analysis

Statistical analysis was performed using SAS^®^ system, PC release 9.2 (SAS Institute, Cary, US). This was a pilot study and a formal sample size calculation was not feasible. In the previous absorption study (13) a sample size of four patients for each dose regimen was able to show the gastrointestinal absorption of the T3S and the extent of this; therefore a sample size of 12 patients per dose group was judged sufficient to provide preliminary information about the therapeutic efficacy of T3S and the optimal L-T4/T3S dose ratio.

Primary efficacy variables were TT3, FT3, FT4, TSH. Secondary efficacy variables were T3S, judgment of the patients on L-T4 vs. L-T4+T3S therapy, lipid parameters. Safety variables of the study included vital signs, laboratory parameters (hematology and chemistry) and adverse events. T3S values below the detection limit, 3 ng/dL, were imputed as the limit value 3/√2 after stating that the values above the limit were roughly log-normally distributed. Missing data were not imputed. TSH levels were markedly right-skewed and were therefore log-transformed to obtain approximately normal distributions as required to use parametric statistics. Changes from baseline of laboratory variables and vital signs were analyzed as differences, if the variable distribution was approximately normal and ratios if the variable distribution was approximately log-normal. Conventionally, *p* < 0.05 was considered statistically significant. Means of differences from V1 for T3S, TT_3_, FT_3_, and FT_4_ levels and geometric means of ratios vs. V1 TSH levels (log-normally-distributed) were calculated. For each hormone was first analyzed the change from V1 to V3a, when the effect of the initial T3S dosage was measured in all 36 patients and there was no bias in comparing L-T4+T3S doses due to T3S dose adjustment in poor responders to the initial dosage. Changes (differences or ratios) from V1 to V3a were tested using Student's paired *t* test both overall and within dose groups, and their relationship with T4 dose was examined by one-way ANOVA with T4 dose as an interval variable. The subsequent analysis from V3a to V5 was restricted to the 34 patients who did not require T3S dose adjustment. Mixed-model linear regression analysis of changes (differences or ratios) vs. V1 was used, with visit and dose as fixed effects and subjects as random effects. Visit was always modeled as an interval variable, a one-unit difference representing a 1-month interval between scheduled time of visits. The covariance structure was chosen according to the Akaike criterion corrected for small samples (AICC) as compound symmetry for FT_3_ and FT_4_ and heterogeneous compound symmetry for T3S, TT_3_, and TSH.

## Results

The entire population of 36 patients completed the study. Compliance was assessed on the basis of the tablets consumption, according to the prescribed dose and the treatment duration. All patients followed the scheduled drug regimen, with minimal differences between expected and actual number of returned tablets in only three patients.

Thirty-four patients maintained the same T3S dose from V3a to V5. The T3S dose was increased in 2 patients on the basis of hormonal results: in one patientfrom group A, the T3S dose was increased to 60 μg from V3a to V5; in one patient from group B, the T3S dose was increased to 60 μg from V3a to V3b, and to 80 μg from V3b to V5. The treatment time-frame at stable T3S dose was (mean ± SD) 73.6 ± 6.4 days (min 61–max 87).

**Figure 2:**
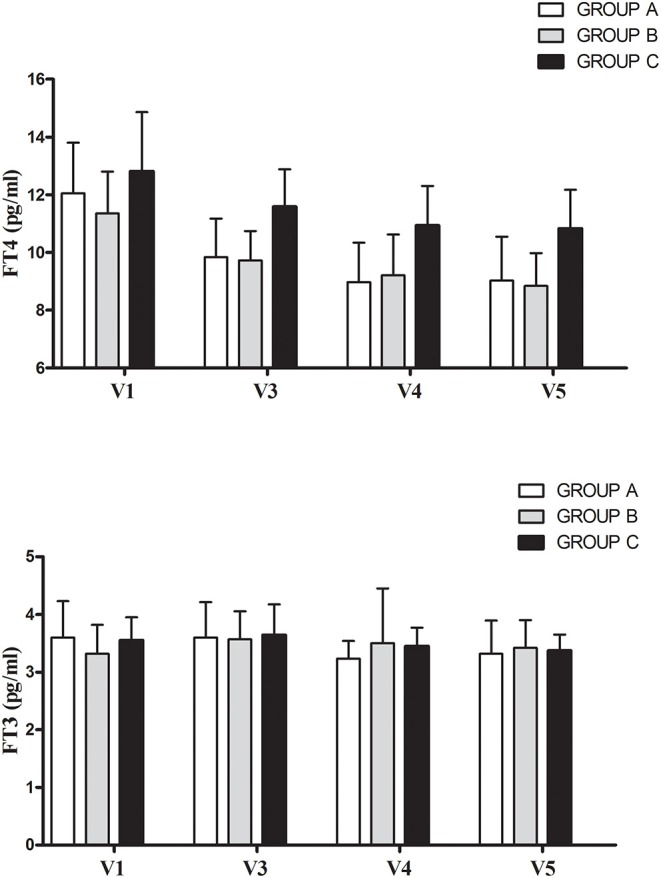
Mean (+ *SD*) changes of free thyroid hormone before (V1) and during L-T4 + T3S therapy in the 34 patients who maintained the same T3S dose throughout the entire study. The various groups are labeled according to the T4/T3S ratio of their treatment regimen: group A = 1.88, group B = 2.5, group C = 3.13.

Table 2 and Figure 2 show FT4, FT3, TT3, TSH, and T3S values measured before and at various time points during T3S administration. A significant, progressive reduction in mean FT4 values was observed, being the largest in the group A and the smallest in group C (*p* < 0.001 in the pooled data), while no relevant variations in FT3 and TT3 values were observed in the three groups. As expected from FT4 reduction, TSH serum levels increased in all groups, the highest value being observed in group A (*p* < 0.001 in the pooled data). T3S levels, measured 24 h after T3S oral administration, remained unchanged throughout the entire study period.

**Table 2:** Serum levels of T3S, TT3, FT3, FT4 (mean and *SD*), and TSH (geometric mean and min-max values) in various groups throughout the study.

	**Visits**	**V1**	**V3a**	**V4**	**V5**
	**Study times (days)**	**0**	**15**	**45**	**75**
	***n***	**36**	**36**	**34**	**34**
T3S ng/dL	Group A	8.68 (3.55)	9.00 (3.55)	8.51 (5.53)	8.98 (4.48)
	Group B	10.55 (4.82)	9.33 (3.34)	13.63 (7.55)	8.55 (3.16)
	Group C	10.89 (5.65)	7.59 (5.22)	6.81 (4.76)	10.04 (7.28)
TT3 ng/dL	Group A	116 (20.1)	122 (11.4)	110 (12.8)	113 (11.5)
	Group B	108 (16.2)	123 (49.9)	111 (17.2)	105 (17.8)
	Group C	116 (15.1)	108 (14.4)	112 (20.4)	112 (21.3)
FT3 pg/mL	Group A	3.61 (0.63)	3.61 (0.61)	3.23 (0.31)	3.32 (0.57)
	Group B	3.32 (0.50)	3.57 (0.48)	3.50 (0.95)	3.42 (0.48)
	Group C	3.55 (0.40)	3.64 (0.53)	3.45 (0.32)	3.37 (0.28)
FT4 pg/mL	Group A	12.05 (1.75)	9.84 (1.33)	8.97 (1.37)	9.03 (1.52)
	Group B	11.35 (1.45)	9.72 (1.02)	9.21 (1.41)	8.84 (1.14)
	Group C	12.81 (2.05)	11.58 (1.30)	10.93 (1.37)	10.83 (1.34)
TSH μUI/mL	Group A	0.382 (0.035–3.520)	0.337 (0.670–2.170)	0.734 (0.157–7.130)	1.954 (0.580–12.700)
	Group B	0.362 (0.027–2.770)	0.365 (0.085–1.550)	0.875 (0.1167–3.370)	1.526 (0.268–7.750)
	Group C	0.402 (0.018–1.160)	0.500 (0.139–1.060)	1.040 (0.244–2.500)	1.050 (0.121–2.310)

**Figure 3:**
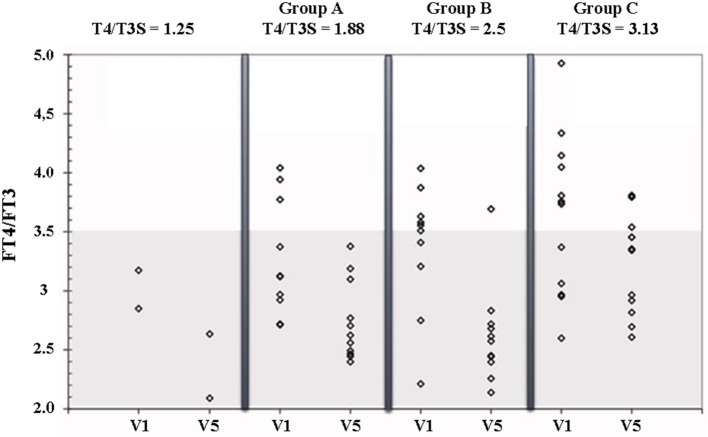
FT4/FT3 ratio in patients on L-T4 treatment (V1) and on L-T4+T3S (V5). The various groups are labeled according to the T4/T3S ratio of their treatment regimen: group A = 1.88, group B = 2.5, group C = 3.13. The 2 patients from group A and group B, who had to increase the T3S dose to 60 and 80 μg, respectively, are indicated as receiving a T4/T3S ratio = 1.25. The shaded area represents the 95% reference range for the FT4/FT3 ratio in the normal euthyroid population.

**Figure 4:**
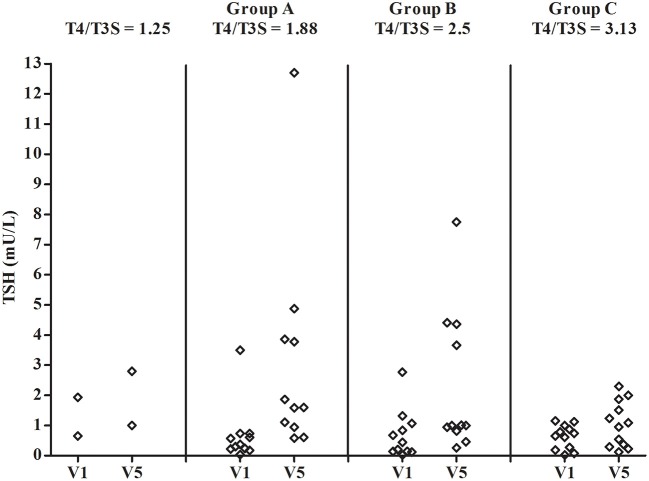
Individual concentrations of serum TSH in patients on L-T4 treatment (V1) and on L-T4+T3S (V5). The various groups are labeled according to the T4/T3S ratio of their treatment regimen: group A = 1.88, group B = 2.5, group C = 3.13. The 2 patients from group A and group B, who had to increase the T3S dose to 60 and 80 μg, respectively, are indicated as receiving a T4/T3S ratio = 1.25.

Figure 3 shows the FT4/FT3 ratio plotted vs the L-T4/T3S dose ratio before and during L-T4+T3S treatment. The 2 patients who had to increase the T3S dose to 60 and 80 μg, respectively, are indicated as receiving a 1.25 L-T4/T3S ratio while the other groups are indicated as receiving 1.88 (group A), 2.5 (group B), or 3.13 (group C) L-T4/T3S ratio. At the start of the study the two patients with the L-T4/T3S dose ratio 1.25 were inside the reference range of the circulating FT_4_/FT_3_ ratio; 4/11 patients of the 1.88 group, 5/11 in the 2.50 group, and 7/12 in the 3.13 group were over the upper limit of the reference range (meaning that 45.4% of the entire population were above the normal range). At the last visit, the FT4/FT3 ratio was within the normal range in all but 1 patient in the 2.5 group and 3 patients in the 3.13 group. Therefore, after combined therapy, the FT4/FT3 ratio was within the normal range in 32/36 patients (88,9%). At V5 serum TSH was above the normal range in 2 patients from group A and one patient from group B (Figure 4).

No correlation was found between the T3S or the L-T4 dose and TT3 or FT_3_ serum levels at the end of the study. Lipid parameters did not show clinically significant changes in all groups.

No T3S-FT_4_-FT_3_-related changes in the safety laboratory tests were recorded. Four patients complained of one adverse event: upper airways inflammation, lumbar pain, chest pain and shoulder fracture due to road accident. No adverse event was judged as related to experimental treatment, and no patient discontinued the treatment.

Both in group A and group B, 5 patients judged the L-T4+T3S treatment better than L-T4 alone while 7 patients did not report differences between the treatments; in group C, the respective numbers were 2 and 10. No patients reported a preference for L-T4 vs. the combined treatment. The reason for preference of L-T4+T3S treatment included physical well-being (12 patients), psychological well-being (9 patients) and tolerability (three patients).

## Discussion

Sulfation of thyroid hormones is catalyzed by sulfotransferases, a family of enzymes located in the cytoplasmic fraction of several tissues, in particular liver, kidney, intestine and brain (5, 15–17). Sulfation of iodothyronines accelerates their deiodination by type I deiodinase (D1) and facilitates their excretion in the bile and urine (18). At the same time, sulfation of T3 protects the active hormone against degradation by the type 3 deiodinase (19). Therefore, while serum levels of T3S are usually very low in the euthyroid adult, they are increased in conditions where type I activity is reduced, such as the fetal life or non-thyroidal illness (20–22). T3S is biologically inactive, but enzymes capable of desulfation of T3 have been described in tissues and in the intestinal miocrobiota, and a potential role of T3S as a reservoir of T3 has been hypothesized under conditions of low D1 activity (23–26). The recent observation that pharmacological administration of T3S can produce steady concentrations of serum T3 suggested that T3S might represent a T3 derivative to be used in combination with T4 in the therapy of hypothyroidism (14).

The results of this pilot study indicate that in hypothyroid patients it is possible to partially substitute L-T4 with T3S, with no reduction of FT3 levels. As a consequence, a normal FT4/FT3 ratio was restored in most patients. The relative amount of administered T4:T3S that better reproduced a physiological hormonal profile was around 3:1. Serum TSH increased, depending on the relative amount of L-T4 that was subtracted from the dose regimen, though remained within the normal range in most patients. This observation suggests that there are no risks of developing thyrotoxicosis after administration of T3S. This consideration is supported by the peculiar metabolism of T3S that is mainly converted to inactive 3,3′-diiodothyronine sulfate by D1. D1 activity is directly regulated by T3 (27). Thus, high T3 levels accelerate T3S deiodination, leaving less T3S available for desulfation. This counter regulatory mechanism would protect against thyroid hormone excess, while promoting the activation of thyroid hormone (desulfation) if the latter is deficient. By the same mechanism, the potential influence of factors affecting D1 activity (e.g., selenium intake) might modulate T3S availability and compensate for changes in T4 to T3 conversion. As expected from the previous study (14), serum T3S, measured 24 h after oral administration, did not show relevant changes due to the rapid clearance of the sulphated iodothyronine. Since we do not have measures of thyroid hormone action in various tissues, we cannot exclude that some biological effects could be exerted in selected tissues capable of local desulfation of the T3S.

One strength of this study is that all patients were thyroidectomized with no residual functioning thyroid tissue to avoid potential interference by endogenous hormonal production. Oral T3S administration did not produce adverse side effects at any of the doses administered, and patients' acceptance appeared favorable, one third judging the L-T4+T3S treatment better than L-T4 alone and the others finding no differences. These results are of difficult interpretation and may be biased because of the unblinded design of the study. Yet, they are reassuring as for tolerability of T3S during a chronic treatment.

Additional limitations of this study include the small number of patients and lack of objective parameters to evaluate thyroid hormone action. Yet, we believe there is sufficient evidence to promote larger studies aimed at evaluating the potential advantages of the combined T4+T3S treatment over T4 monotherapy.

In conclusion, the results of this study indicate that partial substitution of L-T4 with a combination of L-T4+T3S in hypothyroid subjects may allow maintenance of normal levels of serum T3, with restoration of a physiological FT4/FT3 ratio and no appearance of adverse events. Further studies are required to verify whether the chronic LT4+T3S combined treatment of hypothyroidism is able to produce additional benefits over L-T4 monotherapy.

## Data Availability Statement

The datasets generated for this study are available on request to the corresponding author.

## Ethics Statement

The studies involving human participants were reviewed and approved by CEAVNO Comitato Etico Area Vasta Nord Ovest. The patients/participants provided their written informed consent to participate in this study.

## Author Contributions

FS conceived the study design, contributed to data interpretation, and wrote the manuscript. CP, MG, GQ, and IR carried out the patients selection and data collection. GS was involved in the study design and data managing. GC and EG were involved in the data interpretation and writing of the paper. PV contributed to the data interpretation and writing of the manuscript. All authors contributed to manuscript revision, read and approved the submitted version.

### Conflict of Interest

FS, GC, CP, MG, IR, GQ, and PV have researched and received educational grants from Bracco S.p.A. GS is the scientific responsible of Ispharm CRO SRL, granted by Bracco S.p.A, Italy. EG is member of the Fondazione Bracco.
